# Prognostic Value of N1/N2 Neutrophils Heterogeneity and Tertiary Lymphoid Structure in Hepatocellular Carcinoma Patients

**DOI:** 10.1002/cam4.70551

**Published:** 2024-12-24

**Authors:** Yanfei Lang, Weiwei Fu, Wei Xu, Chao Ma, Xiuyun Tian, Chunyi Hao, Shigang Ding

**Affiliations:** ^1^ Department of Gastroenterology Peking University Third Hospital Beijing China; ^2^ Beijing Key Laboratory for Helicobacter Pylori Infection and Upper Gastrointestinal Diseases Peking University Third Hospital Beijing China; ^3^ Key Laboratory of Carcinogenesis and Translational Research (Ministry of Education/Beijing), Department of Hepato‐Pancreato‐Biliary Surgery/Sarcoma Center Peking University Cancer Hospital & Institute Beijing China

**Keywords:** hepatocellular carcinoma, neutrophil, prognosis, secondary follicle‐like TLS, tertiary lymphoid structure

## Abstract

**Background:**

The tumor immune microenvironment, including neutrophils and tertiary lymphoid structures (TLSs), is pivotal for HCC prognosis assessment. Tumor‐associated neutrophils exhibit plasticity, adopting either an antitumorigenic N1 (MPO+ CD206−) or a pro‐tumorigenic N2 (MPO+ CD206+) phenotype. We explored the prognostic value of neutrophil plasticity and TLS maturity in HCC in both tumor and peritumoral tissues and addressed their interaction.

**Methods:**

A retrospective cohort of 79 HCC patients who underwent radical resection from 2015 to 2018 was analyzed, with complete clinical characteristics and survival data of more than 5 years. Multiplex immunohistochemistry identified N1/N2 neutrophils and TLS maturity. Survival differences and correlations with clinical features were assessed.

**Results:**

HCC patients were divided into high‐ and low‐level groups on the basis of the N1 and N2 classifications of neutrophils, revealing a positive correlation with prognosis in tumor tissues and a negative one in peritumoral tissues. TLS maturity stages were associated with prognosis, with a higher proportion of secondary TLS (SFL‐TLS) in peritumoral tissues correlating positively with survival. Further analysis of the correlation between neutrophils and TLSs revealed that most neutrophils infiltrated outside of the TLS in the peritumoral tissues of patients with HCC, and the proportions of SFL‐TLSs and N1 cells in the peritumoral tissue were negatively correlated and positively correlated with survival. Both univariate and multivariate analyses revealed that the N1/N2 ratio in peritumoral tissues was an independent prognostic predictor of HCC.

**Conclusions:**

The N1/N2 ratio of neutrophils and the proportion of SFL‐TLS are considered important prognostic indicators that may reflect the immune microenvironment of HCC patients.

## Graphical abstract

This study highlights the prognostic significance of neutrophils heterogeneity and the maturity of TLSs in HCC in both the tumor and peritumoral tissues. The results indicating that the N1/N2 ratio of neutrophils and the SFL‐TLS proportion in peritumoral tissues are important prognostic predictors in HCC.

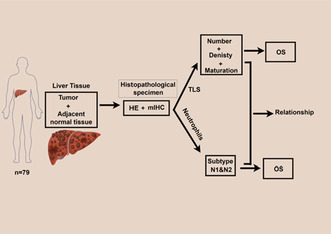

**Abbreviations**
DCs: dendritic cells
HCC: hepatocellular carcinoma
HR: hazard ratio
ICIs: immune‐checkpoint inhibitors
mIHC: multiplex immunohistochemistry
N: neutrophil
NLR: neutrophil‐to‐lymphocyte ratio
OS: overall survival
PFL‐TLS: primary follicular TLS
SFL‐TLS: secondary follicle‐like TLS
TAMs: tumor‐associated macrophages
TANs: tumor‐associated neutrophils
TLSs: tertiary lymphoid tissues
TME: tumor immune microenvironment

## 1 Introduction

HCC is a leading cause of cancer‐related mortality, with limited systemic therapy options and a propensity for early postoperative recurrence and metastasis [1]. Immunotherapy, particularly the combination of immune‐checkpoint and VEGF inhibitors, has shown promise but faces challenges because of patient non‐responsiveness [2]. Thus, understanding the tumor immune microenvironment (TME) is crucial for identifying prognostic markers and improving treatment outcomes.

The immune microenvironment of HCC is mainly composed of tumor‐associated macrophages (TAMs), neutrophils, dendritic cells (DCs), tumor‐infiltrating lymphocytes, and so on [3]. In the past decade, it has become increasingly clear that neutrophils are involved in tumor‐related inflammation, thereby driving disease progression and metastasis [4]. Neutrophils are an important component of the TME and have attracted more attention in recent years. In the vast majority of human solid tumors, high infiltration of neutrophils is associated with accelerated tumor growth, lymph node metastasis, and poor overall prognosis [5]. Neutrophils, with their plasticity, contribute to tumor progression and metastasis through pro‐ and antitumor functions, classified as N1 and N2 phenotypes, respectively [6]. Until now, the plasticity and prognostic value of tumor‐associated N1 and N2 neutrophils in HCC remain largely unknown.

Tertiary lymphoid tissues (TLSs) are the ectopic lymphoid tissues formed during the response to chronic inflammation or persistent immune stimulation. TLSs are associated with better prognosis and immunotherapy response in solid tumors, including HCC [7]. Most studies have found that the presence of TLSs was a positive prognostic factor for HCC patients [8], whereas Finkin et al. [9] believe that the presence of TLSs is a negative prognostic factor. TLS can be further classified into three maturation stages, early‐TLS (E‐TLS), primary follicle‐like TLS (PFL‐TLS), and secondary follicle‐like TLS (SFL‐TLS), on the basis of their cellular composition. Secondary lymphoid follicles (SFLs) represent a distinct type of tertiary lymphoid structure characterized by the co‐expression of CD3+, CD20+, CD21+, and CD23+ cells. They are composed of a germinal center, which is rich in proliferating B cells, surrounded by a mantle zone containing resting B cells, T cells, and follicular dendritic cells. These structures play a pivotal role in adaptive immunity by facilitating the activation, differentiation, and maturation of B and T cells in response to antigens [10]. In 2020, Xu W et al. identified, using spatial transcriptomics and multispectral fluorescence analysis, that SFL‐TLS structures are enriched with mature plasma cells producing high levels of immunoglobulins. The clear cell renal cell carcinoma patients with SFL‐TLS demonstrated enhanced immunotherapy responses and prolonged survival [11]. However, the prognostic value of different TLS maturity statuses in HCC remains unclear.

Lastly, HCC is a typical type of inflammation‐driven cancer characterized by cirrhotic nodules that are rich in TLSs in non‐tumor tissue [12]. Previous studies have long been focused on the immune microenvironment of tumors, with significantly less attention given to the peritumoral tissues. The peritumoral tissue is a unique intermediary interface between the neoplastic and the neighboring healthy tissue [13]. Unique immune signatures are present in the peritumor compared with the tumor and healthy tissue [14]. During neoplastic tissue growth, microenvironment aberrations arise in the peritumor, and investigations of this profile are contributing to advances in cancer diagnosis and prognosis [15]. However, the composition and characteristics of TLS and neutrophils in peritumoral tissues and their relationship with prognosis remain to be clarified.

In this paper, a retrospective cohort survival analysis in 79 HCC cancer and paired peritumoral tissues were used to comprehensively investigate the prognostic value of neutrophils heterogeneity and TLSs. We found that the prognosis of patients was negatively correlated with N1 neutrophils and N1/N2 ratio in peritumoral tissues, whereas positively correlated with N1/N2 ratio in the tumor. Moreover, the high SLF‐TLS that had active germinal centers (GCs) in peritumoral tissues indicated a more favorable prognosis. Additionally, the correlations between neutrophils and TLS showed the proportion of SFL‐TLS and N1 neutrophil numbers in the peritumoral tissue were negatively correlated and positively correlated with survival. Further relationship analysis between the clinical features and neutrophils heterogeneity indicated that N1/N2 ratio in peritumoral tissues could serve as an independent prognostic predictor of HCC.

## 2 Material and Methods

### 2.1 Patients Sample Collection

We retrospectively enrolled patients with HCC diagnosed by surgical pathology at Peking University Cancer Hospital from December 2015 to December 2018. All patients were treated with radical surgery and followed up until December 30, 2023. The following patients were excluded: (I) with comorbidity of severe diabetes mellitus, heart failure, liver, and/or kidney failure; (II) have a history of schizophrenia; (III) have a history of other malignancies or postoperative detection of metastatic liver tumors; (IV) dead during surgery in the hospital; (V) has another organ removed during surgery; and (VI) special populations, such as pregnant and lactating women. In total, we enrolled 79 patients and collected both the tumor and peritumoral tissues. The peritumoral tissue was obtained at a distance of more than 1 cm from the tumor edge, and there was no tumor visible under the microscope assessed by two experienced pathologists independently. Written informed consent was obtained from all patients before the study was conducted. This study was approved by the Institutional Review Board of Peking University Cancer Hospital (approval number: 2015KT72).

### 2.2 Baseline and Laboratory Parameters

Clinical and lab data of 79 HCC patients were collected pre‐surgery and followed every 3 months for the first 2 years after surgery and subsequently every 6 months until death.

### 2.3 H&E and Tertiary Lymphoid Structures Calculation

Slides underwent dewaxing, hematoxylin and eosin staining, and imaging with Aperio ImageScope v12.1 and Hamamatsu Panoramic Scanner (NanoZoomer S60). Two experienced pathologists independently assessed the slides for HCC tissue or peritumoral tissues on the basis of H&E staining. The number of dense lymphocytic aggregates was quantified per 10× high‐power field (HPF) in all hematoxylin and eosin (H&E)‐stained diagnostic sections from the tumor or peritumoral controls. Five fields were randomly selected for TLS counting and then averaged for statistical analysis. The TLS density was calculated as the number of TLSs per mm^2^. TLSs were counted per 10× field. Diameter (d) = 0.22 mm, S = p1/4d^2^ = 3.8 mm^2^. TLS density = Total of 5 random views/5/S, as described previously [16]. TLS characterization and quantification [17, 18]: To quantify TLS abundance, whole slide images were divided into intra‐tumoral and peri‐tumoral regions. TLS scoring for the intra‐tumoral region included four categories: (0) no TLS; (1) 1–2 TLS; (2) 3 TLS; and (3) 4 or more TLS. For the peri‐tumoral region, scoring was as follows: (0) no TLS; (1) TLS present in < 25% of the area; (2) TLS in 25%–75% of the area; and (3) TLS in > 75% of the area.

### 2.4 Immunohistochemistry (IHC)

Liver cancer tissue sections were incubated, dewaxed, and rehydrated. Peroxidase activity was blocked, and antigen retrieval was performed. The sections were then blocked and incubated with primary antibodies overnight at 4°C. Following this, secondary antibodies were applied, and detection was carried out using 3,3′‐diaminobenzidine (DAB) staining. The staining intensity across multiple fields of view was quantified using ImageJ software.

### 2.5 The mIHC Staining and Analysis

Upon TLS identification in H&E‐stained slices, multi‐IHC staining for CD3, CD20, CD21, and CD23 was performed, following a detailed protocol to characterize lymphocyte aggregates in tumor and peritumoral tissues. TLSs were identified as lymphocyte aggregates with histological features similar to those of B cells (CD20, 1:200, ab78237, Abcam, USA), T cells (CD3, ab16669, Abcam), follicular DCs (FDCs) (CD21, 1:100, ab227668, Abcam) and germinal center (GC) cells (CD23, antibody working solution, ZM‐0273) in lymphatic tissue in tumor or peritumoral tissue [19].

For TLS maturity analysis, slides stained for CD3, CD20, CD21, and CD23 were examined using the Inform 3.0 Imaging System. Opal PolarisTM 7‐color artificial mIHC kits were used to detect tyramide via the signal amplification method. TLS stages were classified on the basis of the presence of specific markers: Early (E‐TLSs) lacked FDCs and GCs; primary follicle‐like (PFL‐TLSs) had FDCs; and secondary follicle‐like (SFL‐TLSs) showed FDCs and GCs [16].

For multicolor immunofluorescence staining, slides were stained with MPO (1:4000, ab208670, Abcam), CD206 (1:4000, ab64693, Abcam), and DAPI, using Opal fluorescent dyes and microwave method for antibody removal. The Inform 3.0 Imaging System captured multispectral HPF images to classify tumor‐associated neutrophils as N1 (MPO+ CD206−) and N2 (MPO+ CD206+) [20, 21].

### 2.6 The External Database Cohort Analysis

Single‐cell RNA‐seq data (GEO accession GSE149614; [22]) were processed using Harmony to remove batch effects, followed by clustering and subclustering with Seurat [23]. N1 and N2 neutrophils were annotated separately from myeloid cells, with other annotations based on the original study. A total of 71,915 cells from tumor of 10 HCC patients were analyzed. Differentially expressed genes were identified using FindAllMarkers (avg_log2FC > 0.25, *p*‐value < 0.01), with the top 100 genes from each cell type selected. Bulk RNA‐seq data from TCGA, comprising 367 HCC tumors, were used to assess immune infiltration via CIBERSORT. The N1/N2 ratio was computed with a 0.001 adjustment for negligible N1 values. We then analyzed tumor microenvironment (TME) cell infiltration and overall survival differences across differentially expressed genes (DEG)‐based consensus clusters. Prognostic DEGs were characterized using principal component analysis (PCA), and a TLS score formula was derived: TLS score = ∑(PC1 + PC2), where PC1 and PC2 represent DEG expression in two dimensions, reflecting individual TLS levels [24]. Kaplan–Meier curves and log‐rank tests assessed the impact of N1/N2 ratios and TLS scores on overall survival. A Cox regression model incorporating N2 proportions and N1/N2 ratios was developed to predict survival at multiple time points.

### 2.7 Statistical Analysis

Statistical analyses were conducted using R and SPSS, with packages for optimal cutoff determination. Continuous variables were compared using *t*‐tests or *U* tests, and relationships assessed with Pearson's *χ* ^2^ or Fisher's tests. Spearman's analysis evaluated correlations. OS was evaluated via Kaplan–Meier and log‐rank tests, with Cox analyses identifying predictors (HR and 95% CI). The cutoff values were determined using the R packages (survival and survminer). Significance was set at *p* < 0.05.

## 3 Results

### 3.1 Demographic Data and Clinical Characteristics

Retrospective study of 79 primary HCC patients (Figure S1), median age 59, 71 male:8 Female, identified main risk factors: HBV, HC, and alcohol. No preoperative therapy. 44‐month median follow‐up revealed 88.9% perineural invasion. The baseline characteristics were displayed in Table S1.

### 3.2 The Distribution and Composition of Neutrophils in Tumor and Peritumoral Samples of HCC

**FIGURE 1:**
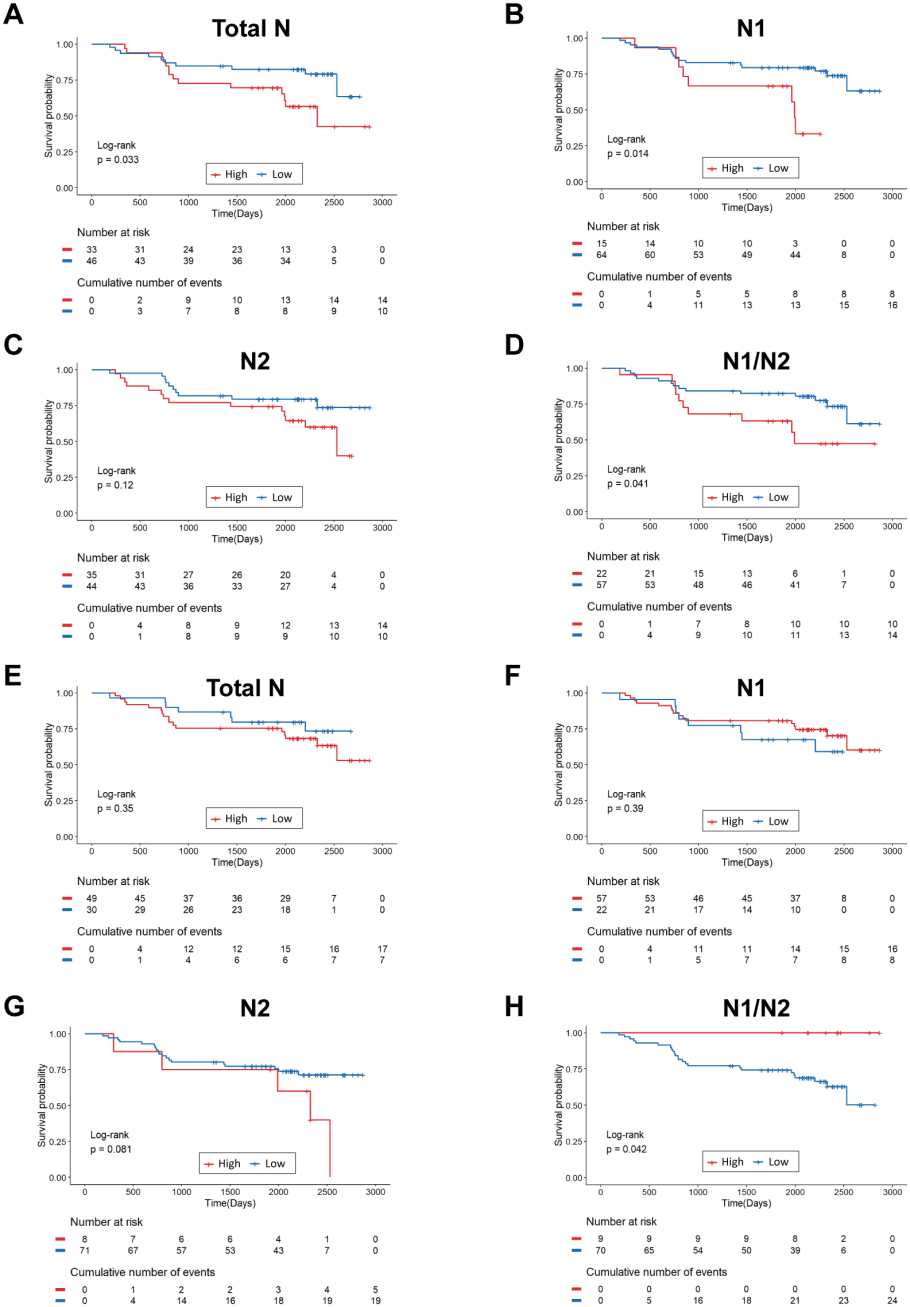
Kaplan–Meier and log‐rank tests revealed the predictive value of neutrophils for HCC prognosis. (A–D) Kaplan–Meier OS plots by neutrophil counts in HCC peritumor. (E–H) Kaplan–Meier OS plots for HCC tumor neutrophils.

Because neutrophils have differential states of activation/differentiation, suggesting that they can convert to “N1‐phenotype” (MPO+ CD206−) or “N2‐phenotype” (MPO+ CD206+) state, as shown in Figure 1A and Figure S2 [21, 25]. The median number of N1 and N2 neutrophils in TME were 72 and 5.6, respectively (Table S2). The median number of N1 and N2 neutrophils in peritumoral tissues were 61 and 5, respectively (Table S3).

**FIGURE 2:**
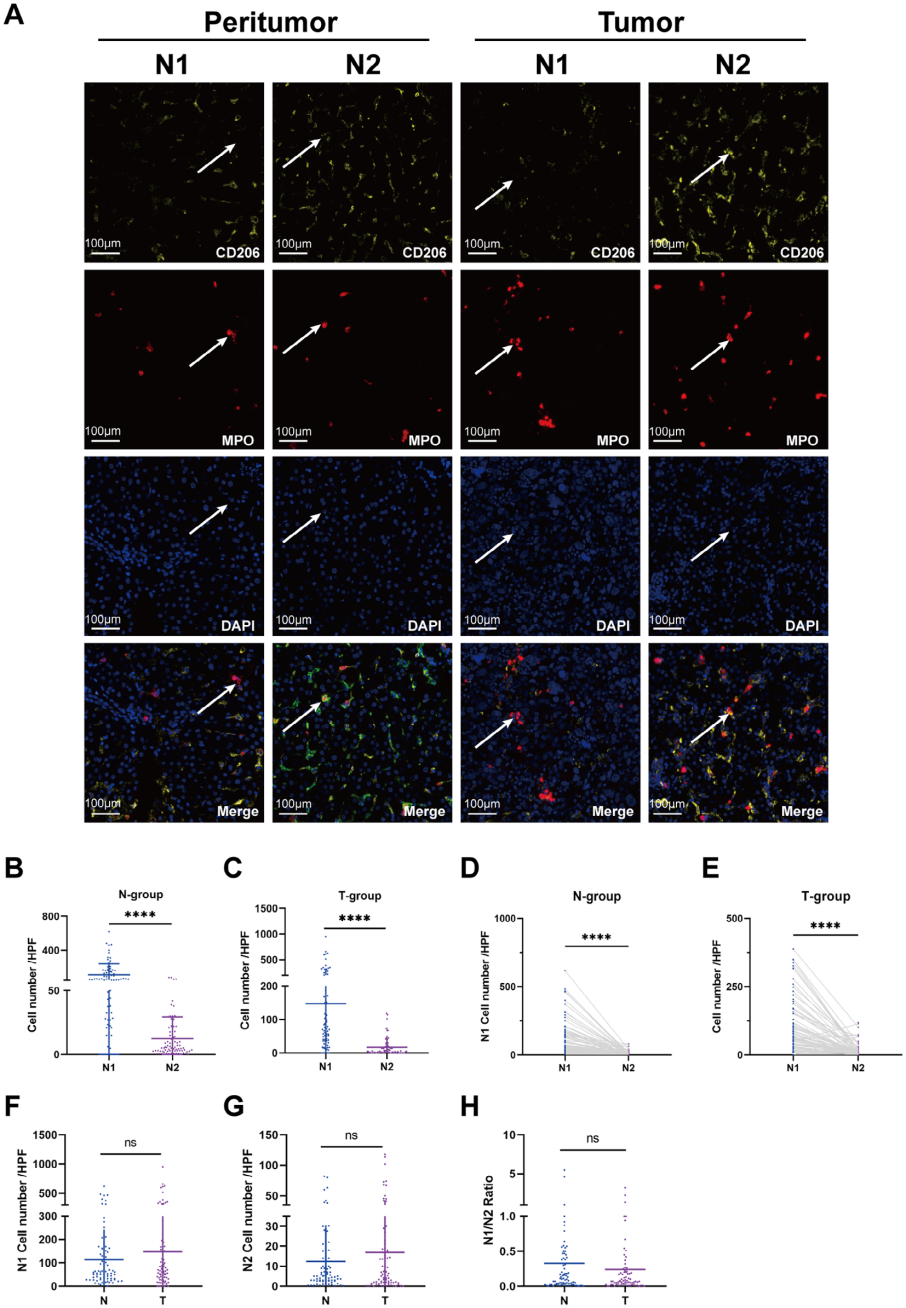
Identification of N1 and N2 neutrophils within HCC peritumor and tumor tissues. Representative images of IF staining for N1 and N2 (white arrows) for the tumors. (B, C) Unpaired analysis of N1, N2 neutrophils in HCC peritumor and tumor tissues. (D, E) Paired analysis of N1 and N2 neutrophils in HCC peritumor and tumor tissues. (F–H) Unpaired analysis of N1, N2, N1/N2 in HCC peritumor (*n* = 79) and tumor tissues (*n* = 79) by unpaired analysis. N‐group: Peritumoral tissues; T‐group: Tumor tissues. ****p < 0.0001.

In peritumoral tissues, N1 neutrophils averaged 114.0, exceeding N2's 12.4 (*p* < 0.0001, Figure 2B). In tumors, N1 also dominated at 147.7 over N2 (*p* < 0.0001). Paired analysis confirmed these differences (*p* < 0.0001, Figure 2C–E). However, the unpaired analysis showed no significant difference in N1 neutrophils (Figure 2F), N2 neutrophils (Figure 2G), and N1/N2 ratio (Figure 2H) between tissues. Both tumoral and peritumoral tissues show N1 neutrophil predominance with no compositional difference.

### 3.3 The Relationship Between Neutrophils Composition and the Prognosis of HCC Patients

Tissue study categorized patients by N1 neutrophil count and ratio; sample sizes and clinical correlations detailed in Tables S1 and S4. Then, we analyzed the correlation between the high and low groups of each indicator and the prognosis of patients. We found in peritumoral tissues that low neutrophil counts and N1/N2 ratio correlated with better survival (*p* < 0.05) (Figure 1A,B,D). Still, they had no significant relationship with N2 neutrophils (Figure 1C). However, better overall survival was associated with higher N1/N2 ratio (*p* < 0.05) in TME (Figure 1H), but not with the total number of tumor‐associated neutrophils, N1 and N2 number (*p* > 0.05) (Figure 1E–G). Thus, the N1/N2 ratio inversely linked to prognosis in peritumoral tissue but positively in tumor environment. Similarly, results from TCGA database showed that a higher N1/N2 ratio in tumor tissue was associated with better prognosis (Figure S3A–C).

### 3.4 The Presence of TLS and HCC Patient's Survival

TLSs were presented in many solid tumors and aggregated lymphocytes were typical [26]. Firstly, the histological TLS prognosis assessed by H&E analysis (Figure S4A). Pathological examination showed TLS in 65 peritumor (82.3%) and 29 tumor (36.7%) tissues. Then, the analysis of TLS count and density showed higher values in peritumoral areas versus cancer tissue, with significant stats. (Figure S4B,C). To verify the prognostic value of TLS: R packages set cutoff, divide into positive/negative groups. As shown in Figure S4D,E, no significant survival differences in TLS‐negative versus TLS‐positive peritumoral and tumoral tissues. Further, determined TLS number/density cutoffs by R packages; grouped peritumoral and tumoral tissues into TLS‐low and TLS‐high by TLS area density (Figure S4A). The numbers of high/low groups of TLSs in tissues were shown in Table S3. The results showed that higher TLS counts linked to better survival in peritumoral tissues (*p* < 0.05, Figure S4F), not density (Figure S4H). Our TLS results in tumor tissues show a trend of positive correlation with prognosis (Figure S4G,I). Combined with the TCGA analysis, our results suggested that TLS in tumor tissue is positively correlated with survival (Figure S3D).

### 3.5 Characteristics of TLS Maturation and Cellular Components Heterogeneity in HCC

**FIGURE 3:**
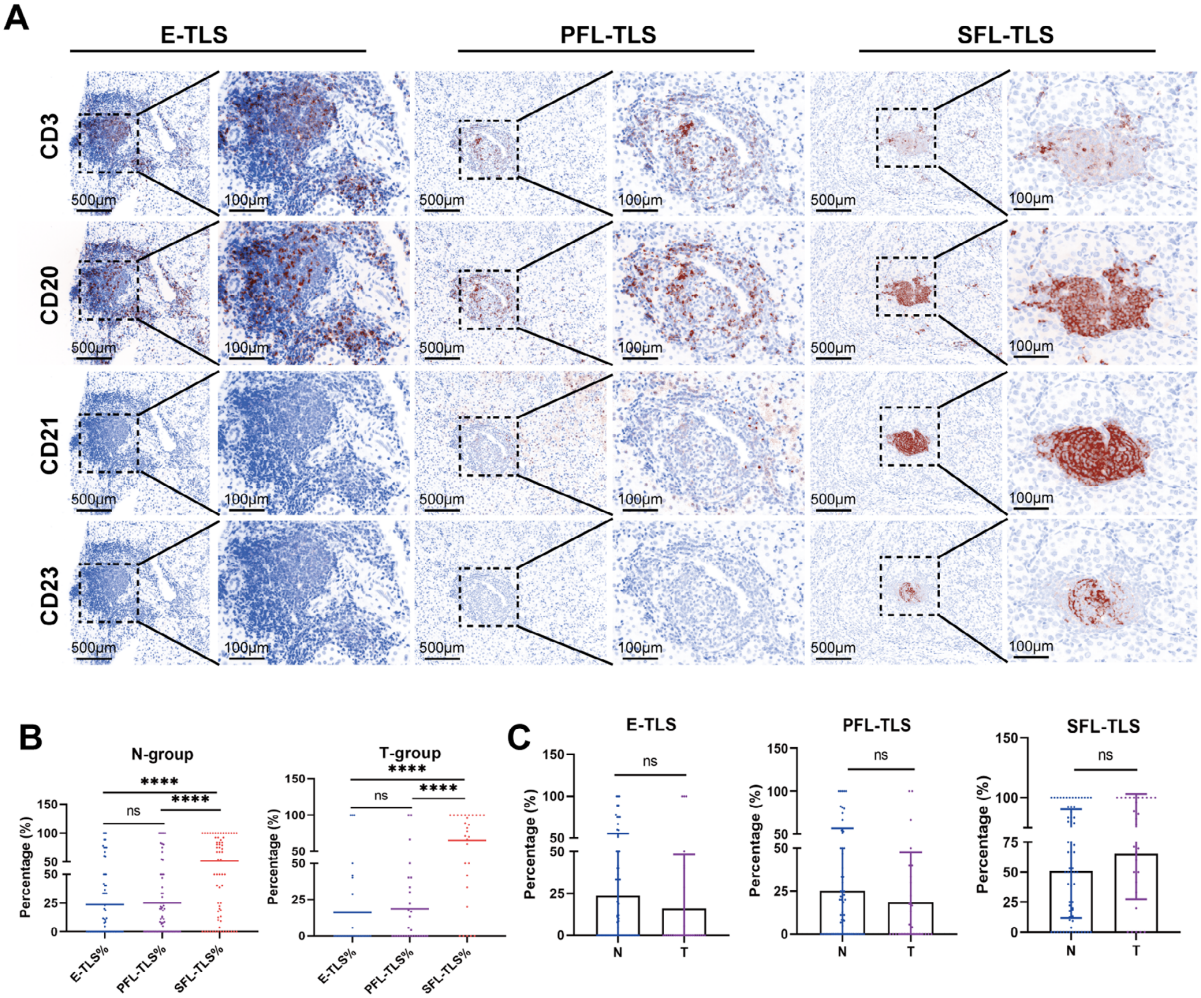
TLS maturation status in HCC peritumor and tumor tissues. IHC assessed lymphocyte aggregates in HCC tissues, identifying TLS stages via CD markers (*n* = 79). N‐group: Peritumoral tissues; T‐group: Tumor tissues. E‐TLSs: Early TLS lacked FDCs and GCs; PFL‐TLSs: Primary follicle‐like TLS had FDCs; SFL‐TLSs: Secondary follicle‐like TLS showed FDCs and GCs. Unpaired *t*‐tests compared TLS stages in peritumor vs. tumor tissues. ns, not significant; ****p < 0.0001.

The TLS maturation stages linked to cancer prognosis [16]. Therefore, understanding TLS heterogeneity—cellular makeup, location, maturation, and effects on antitumor immunity—is crucial. mIHC assay was used to assess TLS maturation in HCC, identifying lymphoid tissue‐like structures with CD3+, CD20+, CD21+, and CD23+ cells. Multistage TLS maturation was observed (Figure 3A). Investigated TLS maturation in HCC tissues (Figure 3B) showed SFL‐TLS predominates in both peritumoral and tumor tissues (*p* < 0.0001), with similar proportions across groups (Figure 3C), indicating SFL‐TLS was the most common in both, without significant group differences.

### 3.6 The Association Between TLS Maturation Status and HCC Prognosis

We further examined TLS maturity's link to clinical outcomes, including samples with TLS. We set thresholds for E‐TLS, PFL‐TLS, and SFL‐TLS percentages, categorizing subjects into low and high groups. TLS% distribution in tumor and peritumoral tissues detailed in Table S5. Patients were categorized by TLS% thresholds. Clinicopathological correlations for PFL% and SFL% in normal and tumor tissues are summarized in Table S6.

**FIGURE 4:**
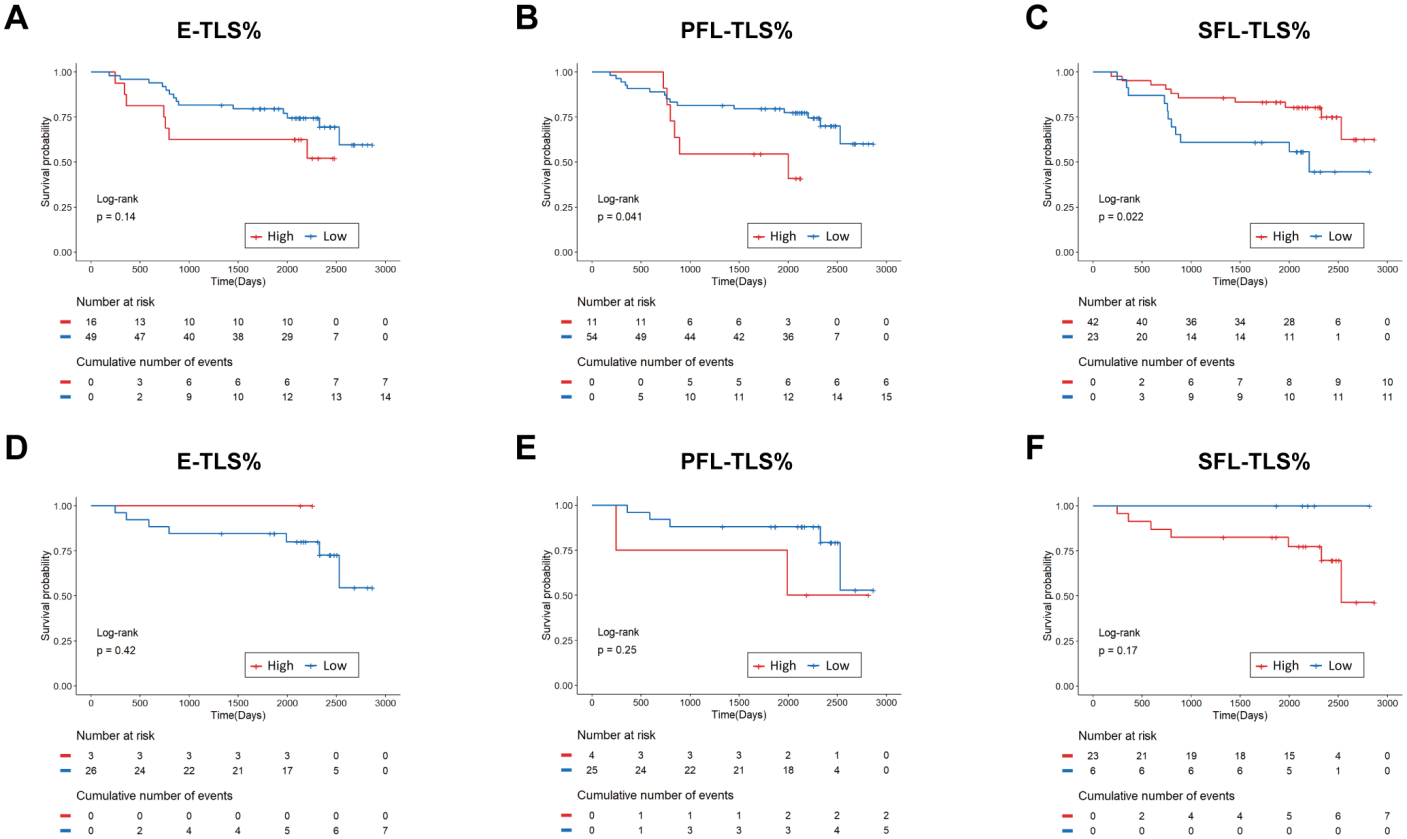
Kaplan–Meier and log‐rank tests revealed the predictive value of TLS maturation. (A–C) Kaplan–Meier plots for OS according to the proportions of different TLS maturation stages in the peritumoral region. (D–F) Kaplan–Meier plots for OS according to the proportions of different TLS maturation stages. E‐TLS%, proportion of E‐TLSs; PFL‐TLS%, proportion of PFL‐TLSs; SFL‐TLS%, proportion of SFL‐TLSs.

Subsequently, we assessed TLS maturation's prognostic impact in HCC. In peritumoral tissues, lower PFL‐TLS% and higher SFL‐TLS% correlated with better survival (*p* < 0.05), unlike E‐TLS% (*p* > 0.05). No TLS%‐survival link in tumors (Figure 4). These above results indicated that SFL‐TLS% positively predicted prognosis, but tumor TLS maturity did not.

### 3.7 The Neutrophil Heterogeneity Correlated to the TLS Distribution and Maturation

**FIGURE 5:**
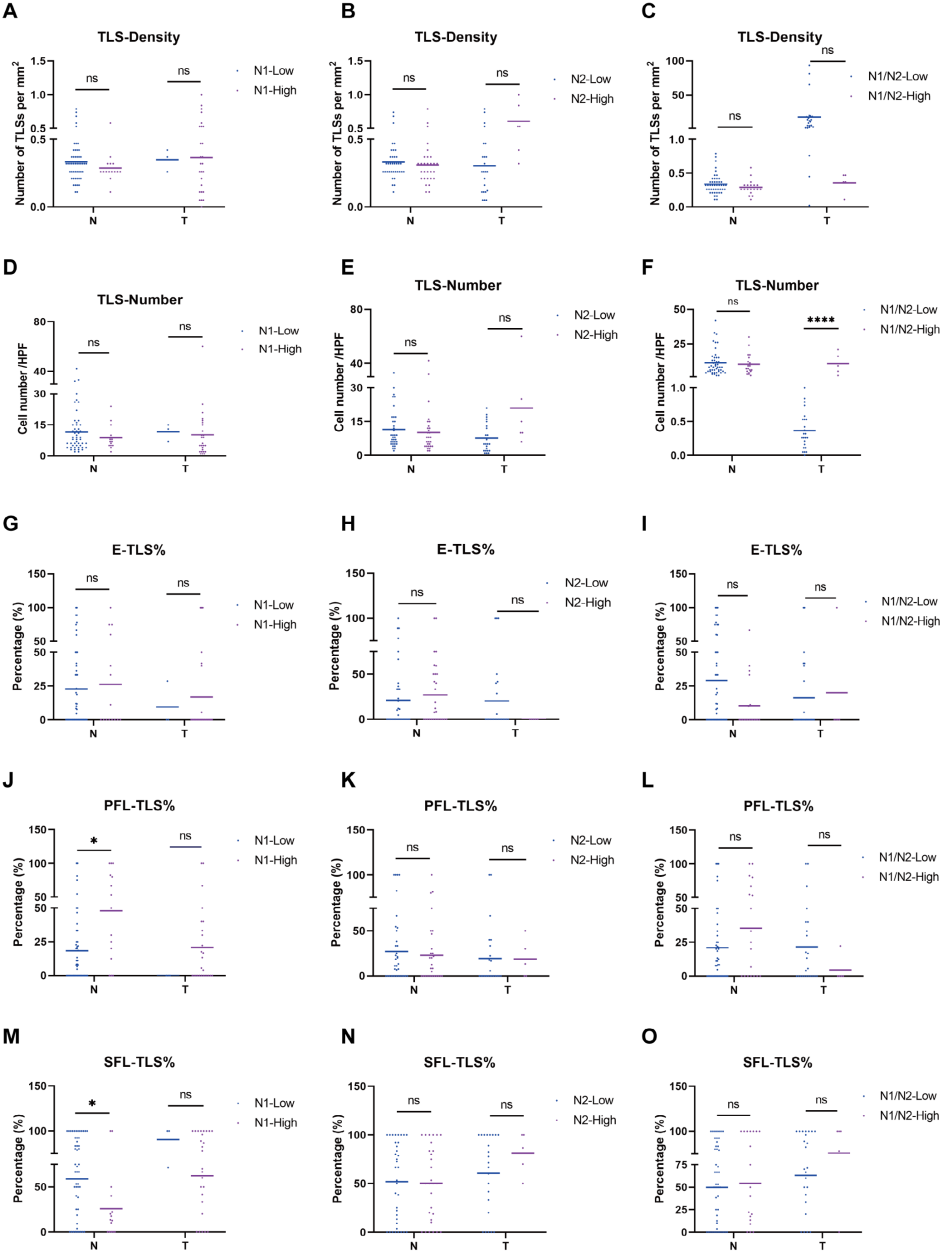
Correlation analysis of neutrophils and TLSs in peritumoral and tumor tissues. (A–C) TLS density comparison in HCC tissues from different N1/N2 ratios. (D–F) TLS count differs among neutrophil groups in HCC. (G–I) E‐TLS proportions vary by N1, N2, N1/N2 in HCC. (J–L) PFL‐TLS ratios by N1, N2, and N1/N2 in HCC tissues. (M–O) SFL‐TLS distribution by N1, N2, and N1/N2 in HCC. ns, not significant; *p < 0.05.

Many studies have shown that NLR with TLS analysis predicts tumor prognosis. Thus, we detailed neutrophil infiltration's link to local TLS, analyzing TLS metrics across N1/N2 groups (Figure 5A–F). These results suggested that high N1/N2 ratio linked to more TLS in tumors (Figure 5F). High N1 count reduced SFL‐TLS%, increased PFL‐TLS% in peritumoral tissues (Figure 5G–O). N1 and PFL‐TLS% negatively, SFL‐TLS% positively correlated with prognosis, suggesting neutrophil heterogeneity influences TLS distribution and immune responses.

### 3.8 The Localization and Maturation Status of TLS Were Associated With Neutrophil Heterogeneities

**FIGURE 6:**
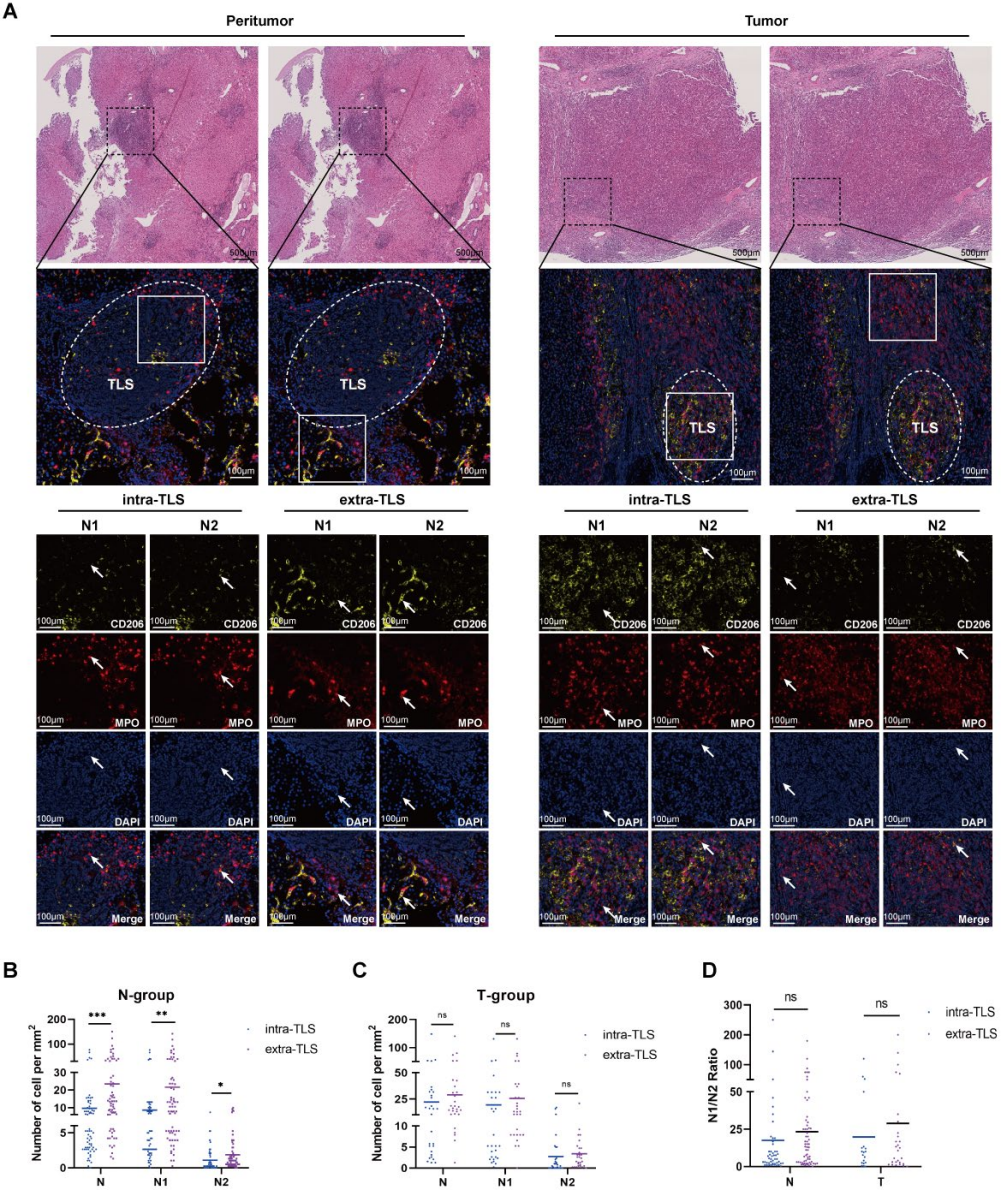
The distribution of neutrophils intra‐ and extra‐TLSs in peritumor and tumor tissues. (A) N1, N2 neutrophils in/out TLS in HCC tissues. The white circle: TLS; the white square showed neutrophils inside/outside TLS. (B) Neutrophil count comparison in peritumor TLS groups. (C) Neutrophil count in tumor intra/extra‐TLS groups. (D) N1/N2 ratio in intra/extra‐TLS groups in tissues. N‐group: Peritumoral tissues; T‐group: Tumor tissues. ns, not significant; *p < 0.05, **p < 0.01, ***p < 0.001.

TLSs are thought to be crucial for tumor/peri‐tumoral immune cell interactions, including anti and pro‐tumor types [27]. Thus, neutrophil localization and heterogeneity in and out of TLS were detailed (Figure 6A). Total and N1/N2 neutrophil counts were higher outside TLS in peritumoral tissue (Figure 6B), with no N1/N2 ratio difference (Figure 6D), suggesting neutrophils predominantly reside outside TLS with similar compositions. No significant intra−/extra‐TLS differences in tumor tissues (Figure 6C,D).

**FIGURE 7:**
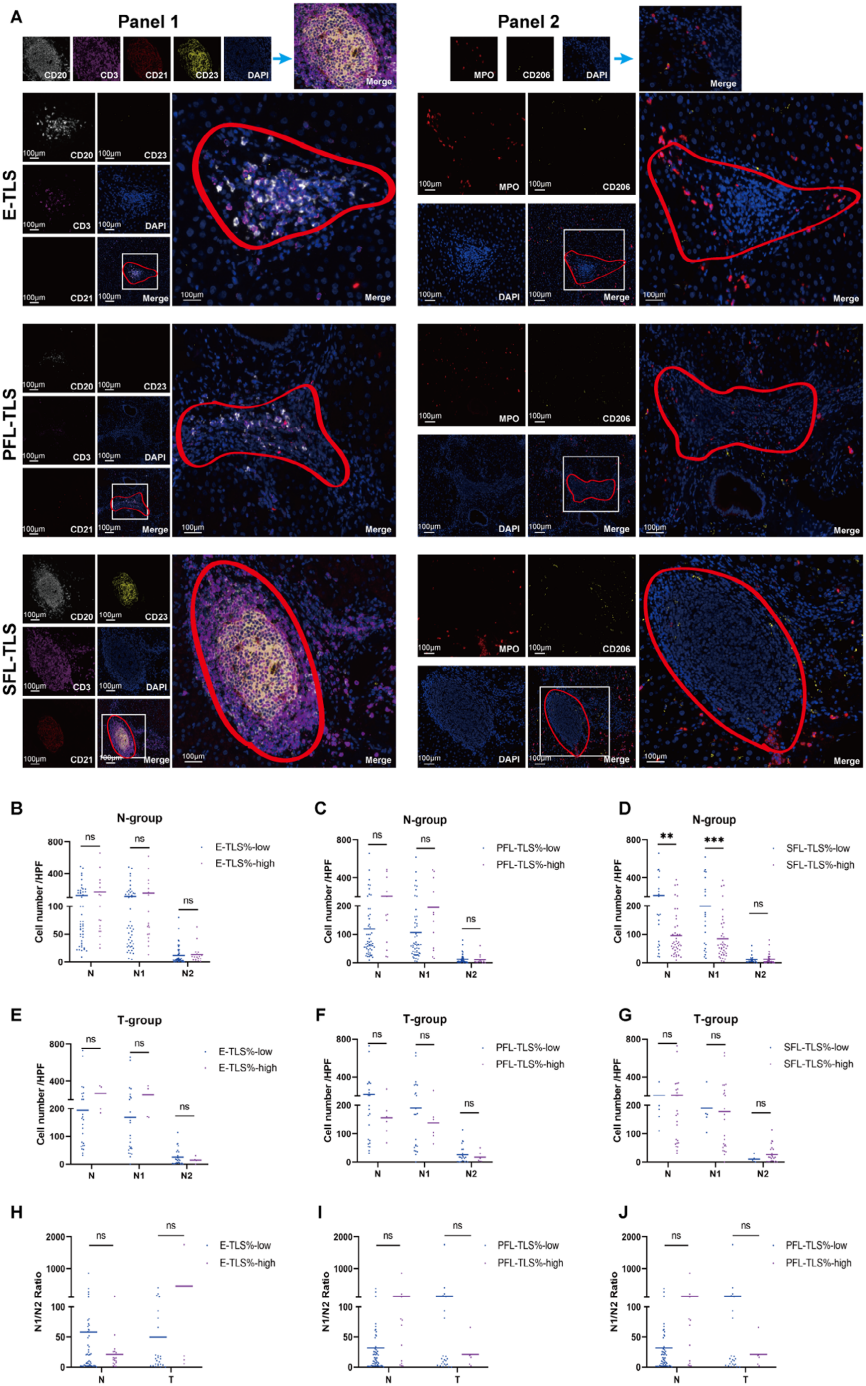
Correlations between TLS maturation and the number of neutrophils in peritumor and tumor tissues. (A) mIHC analyzed TLS and neutrophil relations in HCC tissues. Left panel: Red circle = tertiary lymphatic area; white square = enlarged view. Right panel: Red circle = TLS area; white square = neutrophil infiltration. (B–D) Neutrophil counts by E‐TLS%, PFL‐TLS%, SFL% in peritumor. (E–G) Neutrophil counts by E‐TLS%, PFL‐TLS%, SFL% in tumor. (H–J) N1/N2 ratios in E‐TLS%, PFL‐TLS%, SFL% groups. N‐group: Peritumoral tissues; T‐group: Tumor tissues. ns, not significant; **p < 0.01, ***p < 0.001.

The association of TLS number and density on the composition of infiltrating neutrophils in both the tumor and peritumoral tissues was further analyzed. As shown in Figure S5, TLS number/density unrelated to neutrophil composition in tumor/peri‐tumoral tissues. Further explored TLS maturity's link to neutrophil composition (Figure 7). As mentioned above, divided population by TLS% thresholds; high SFL% group had fewer total and N1 neutrophils in peritumoral tissues than low SFL% (Figure 7D). This, with prior findings (Figure 5M), indicates negative correlation between SFL% and N1 neutrophils, positive with survival (Figures 1B and 4C). No difference in neutrophil counts across TLS maturity stages in tumors.

### 3.9 The N1/N2 Ratio in Peritumoral Tissues Was an Independent Prognostic Predictor of HCC

Finally, we demonstrated the relationship between the N1 and N2 neutrophils, PFL%/SFL‐TLS%, and clinical features. As shown in Figure S6A, N1/N2 ratio was negatively correlated with CEA in peritumoral, not tumoral tissues. Optimal cutoffs for PFL‐TLS% and SFL‐TLS% in peritumoral tissues were 53.3 and 23.0, classifying 54 and 23 patients as low groups. As shown in Figure S6B, PFL‐TLS% was positively correlated with ALT, AST, and CA199 in peritumoral. In addition, PFL‐TLS% was linearly correlated positively with CA199 and AST; SFL‐TLS% was negatively with AST (Figure S6C).

Further correlation analysis between neutrophils, TLS, and clinical prognostic parameters showed smoking, tumor size, vascular invasion, TMN stage, AST, TCHO, LDL‐C, GGT, and N‐N1/N2 ratio significantly associated with OS in univariate Cox analysis (Table S7). Notably, the correlation of the N1/N2 ratio with survival was consistent with the results from the TCGA analysis (Figure S3E). Multivariate analysis confirmed liver capsule invasion, GGT, and N‐N1/N2 ratio as independent OS indicators. In TLS‐positive cohort (*n* = 65), univariate analysis linked tumor size, liver capsule invasion, vascular invasion, TMN stage, AST, LDL‐C, GGT, N‐PFL%, N‐SFL% with OS (Table S8). Multivariate analysis reaffirmed liver capsule invasion and GGT as independent OS prognosticators.

## 4 Discussion

The tumor immune microenvironment has become a focal point of cancer research, especially because of its association with prognosis and response to immunotherapy. Although a plethora of studies have explored the role of neutrophil infiltration and TLS in various cancer types, some results are inconsistent [28]. In this paper, we analyzed the heterogeneities of neutrophils and characteristics of TLS in the tumoral and peritumoral tissues of HCC and addressed their prognostic value. Our results showed that N1/N2 ratio was positively associated with prognosis in the tumor microenvironment, while the number of total neutrophils and N1, N1/N2 ratio were negatively associated with prognosis in peritumoral tissue of HCC. Moreover, we also analyzed the correlations between neutrophils and TLS and found that SFL% and N1 numbers in the peritumoral tissues were negatively correlated and positively correlated with survival. Further relationship analysis to the clinical features demonstrated that the N1/N2 ratio in peritumoral tissues was an independent prognostic predictor of HCC.

Neutrophils play a key role in innate antitumor responses, with studies linking them to cancer prognosis [29]. Single‐cell analysis confirmed that targeting tumor‐associated neutrophils for liver cancer treatment [30]. Moreover, NLR in HCC indicates advanced disease and poor outcomes [31]. Despite heterogeneity, CD206 expression differentiates TANs with prognostic significance [29]. This study revealed the N1/N2 ratio's positive correlation with HCC prognosis, aligning with the known antitumor properties of N1 and pro‐tumorigenic traits of N2 in the tumor microenvironment [32].

In contrast, the results in peritumoral tissues showed that N1 neutrophils were the dominant infiltrating neutrophils in peritumoral tissues, and the prognosis of patients was negatively correlated with both N1 and N1/N2 ratio. Consistently, peritumoral neutrophils were reported to have characteristics, functions, and prognostic associations that were different from those of intra‐tumoral neutrophils in HCC, and the high infiltration of neutrophils in the peritumoral tissues was positively correlated with the progression of angiogenesis at the tumor invasion margins in HCC patients [33]. This may be related to the interaction with the microenvironment. In cancerous tissues, neutrophils may have more complex interactions with tumor cells and other immune cells in cancer tissues, such as TAMs and DCs. These interactions could potentially influence the progression and metastasis of the tumor [34]. In peritumoral tissues, neutrophils may help maintain tissue homeostasis by releasing anti‐inflammatory cytokines and participating in immune surveillance [30]. The single‐cell study also showed that neutrophils probably had a dynamic spectrum of pro‐tumor and antitumor functions that vary according to their microenvironment [31]. These indicated that the role of neutrophils in the microenvironment was complex and diverse and might play different or even opposing functions in tumoral and peritumoral tissues. Further understanding of these differences could help in developing new cancer treatment strategies, especially those targeting neutrophils. In addition, our results suggested that more N1 neutrophils might be induced and aggregated in peritumoral tissues for cancers with poorer prognosis. Although N1 neutrophils are generally recognized for their antitumor properties, some works have found that they could exhibit immunosuppressive characteristics under specific conditions. Studies have shown that N1 neutrophils released molecules such as reactive oxygen species, arginase 1, and inducible nitric oxide synthase, which may regulate T‐cell immune function, promote angiogenesis, and tumor growth [35]. N1 neutrophils can express immune checkpoint molecules such as PD‐L1, which by binding to the PD‐1 receptor on T cells, suppress the activity of T cells, leading to immune evasion [36]. The immunological characteristics of N1 neutrophils may depend on the local environment, making them a factor in immune evasion and immunosuppression in the peritumoral area, thereby affecting the prognosis of liver cancer patients. Therefore, more research is necessary to fully understand the specific function of N1 neutrophils in the peritumoral area of HCC.

TLSs are aggregates of immune cells in the TME, and their functions in cancer mainly include promoting the infiltration of immune cells into tumor tissues, activating the antitumor responses of B cells and T cells, and potentially participating in the regulation of immune responses in the tumor microenvironment [37]. The presence of TLS is generally associated with a better prognosis for cancer patients [38]. However, the prognostic role of TLS in HCC is complex and controversial [39]. Some studies have indicated that TLSs within liver cancer tumors are associated with a lower risk of early recurrence, which may reflect sustained and effective antitumor immunity [39]. The presence and maturity of TLS may be closely related to improved clinical outcomes in patients with liver cancer [39]. In contrast, some studies have pointed out that in non‐tumorous liver tissues, TLS may promote the growth of malignant hepatic progenitor cells, suggesting that TLS may have a dual role in liver cancer [39]. In our study, we analyzed the distribution and maturity of TLS in HCC tumors and peritumoral tissues. Firstly, based on the histological evaluation of TLS, we found the presence of TLS was relatively lower in the tumor (36.71%) than in the peritumoral (82.28%). Moreover, we also addressed that better overall survival was associated with higher TLS numbers in peritumor tissues, which is consistent with previous results in other tumors [40]. Our mIHC results showed that SFL proportion was positively correlated with prognosis in peritumoral tissues, which was consistent with previous studies [41]. Many studies have found that SFL‐TLS often predicted a stronger immune response and better prognosis. The main reasons include the following aspects: (1) In the germinal centers of SFL‐TLS, B cell clones are selectively activated and expanded, undergoing class switching and somatic hypermutation. In tumors without SFL‐TLS, B cells are either sparse or differentiate into regulatory cells that produce immunosuppressive cytokines. Notably, tumors with SFL‐TLS, a high density of B cells and plasma cells, and antibodies against tumor‐associated antigens generally have better clinical outcomes and responses to immunotherapy compared with tumors lacking these features [42]. (2) SFL‐TLS possesses the function of enhancing immune surveillance, which helps in the identification and elimination of tumor cells [43]. (3) SFL‐TLS have the function of promoting the infiltration of immune cells, enhancing the local immune response [44]. Thus, the maturity of SFL‐TLS is associated with the prognosis of patients with liver cancer and might be an important factor in predicting the response of liver cancer patients to immunotherapy [45]. Further in‐depth research on regulating TLS can help fully explore its potential application value in the antitumor immune response, guiding the precision immunotherapy of HCC.

In the TME, TLSs are composed of various immune cells, including DCs, B and T lymphocytes. Previous studies have also demonstrated the interaction between TAMs and TLS and their relationship in the occurrence and development of HCC [22]. Though neutrophil infiltration was reported to increase in parallel with TLS density in the peritumoral region [46] and may influence the development and metastasis of tumors through their interactions with TLSs [47]. The relationship between neutrophils and TLSs is still largely unknown. We found that in peritumoral tissues the neutrophils mainly existed outside of the TLS, which was consistent with the results that neutrophils were dominantly infiltrated in the peritumoral region [48]. Additionally, we also found that the N1 number and SFL% in peritumoral tissues were negatively and positively correlated with survival. That was supported by the finding that the neutrophil‐to‐T cell ratio was higher in peritumoral tissue than in the intra‐tumoral tissue and was negatively correlated with the overall survival of patients with HCC [48]. Some studies suggest that tumor‐associated neutrophils may promote lymphatic metastasis of tumors by facilitating lymphangiogenesis and affecting the function of TLS through specific molecular mechanisms, revealing new targets for immunotherapy [49]. Currently, there are few reports on the mechanism of interaction between SFL‐TLS and neutrophils in tumors and even fewer in the peritumoral tissue. Based on existing literature, we hypothesize that the possible interaction mechanisms of neutrophils and SFL‐TLS are as follows: (1) The activation of B cells, class switching, somatic hypermutation at high frequency, and differentiation into plasma cells within mature TLS can produce antibodies against tumor‐associated antigens. These antibodies help to neutralize tumor cells and may enhance the phagocytic action of neutrophils and antibody‐dependent cell‐mediated cytotoxicity [50]. (2) The interaction between neutrophils and TLS may involve a variety of chemokines and cytokines [47]. (3) Neutrophil metabolic pathways, particularly leucine metabolism, may affect their antigen‐presenting functions. Leucine can upregulate the expression of HLA‐DR and related co‐stimulatory molecules in neutrophils, which may be related to immune activation within TLS [51].

In summary, we analyzed the heterogeneities of neutrophils and the characteristics of TLS in the tumoral and peritumoral tissues of HCC and evaluated their prognostic value. We first demonstrated the number of total neutrophils and N1 neutrophils, the N1/N2 ratio was negatively associated with prognosis in peritumoral tissues of HCC, which is different from that in the tumor. In addition, we addressed the N1 number and SFL% in peritumoral tissues were negatively correlated and positively correlated with survival. The N1/N2 ratio in peritumoral tissues was an independent prognostic predictor of HCC. However, there are a few limitations existing in our study. The sample size is relatively small, and this is a retrospective, single‐center study. Future studies with larger sample sizes, as well as prospective cohort studies and multicenter trials are essential to validate the hypotheses proposed in this study. Such studies would be of great value for future clinical applications and prognosis prediction.

## Author Contributions

**Yanfei Lang:** data curation (equal), formal analysis (equal), methodology (equal), software (equal), validation (equal), writing – original draft (equal). **Weiwei Fu:** conceptualization (equal), funding acquisition (supporting), supervision (supporting), writing – review and editing (supporting). **Wei Xu:** data curation (equal), formal analysis (equal), supervision (equal). **Chao Ma:** methodology (supporting), software (supporting). **Xiuyun Tian:** methodology (supporting). **Chunyi Hao:** project administration (equal), resources (equal). **Shigang Ding:** funding acquisition (equal), project administration (equal), writing – review and editing (equal).

## Ethics Statement

This study was approved by the Institutional Reviewer Board, the Beijing Cancer Hospital Medical Ethics Committee (2015KT72).

## Consent

The authors have nothing to report.

## Conflicts of Interest

The authors declare no conflicts of interest.

## Supporting information


Data S1.

## Data Availability

Data supporting the findings of this study are available within the article and its Supporting Information.
