# Supplementary material for: Prognostic Value of N1/N2 Neutrophils Heterogeneity and Tertiary Lymphoid Structure in Hepatocellular Carcinoma Patients
Source: Cancer Med. 2024 Dec 24;13(24):e70551. doi: 10.1002/cam4.70551 (PMC11667523; doi:10.1002/cam4.70551)
Supplement: Supplementary file 1 — Data S1. [file CAM4-13-e70551-s001.docx]

**Fig. S1:**


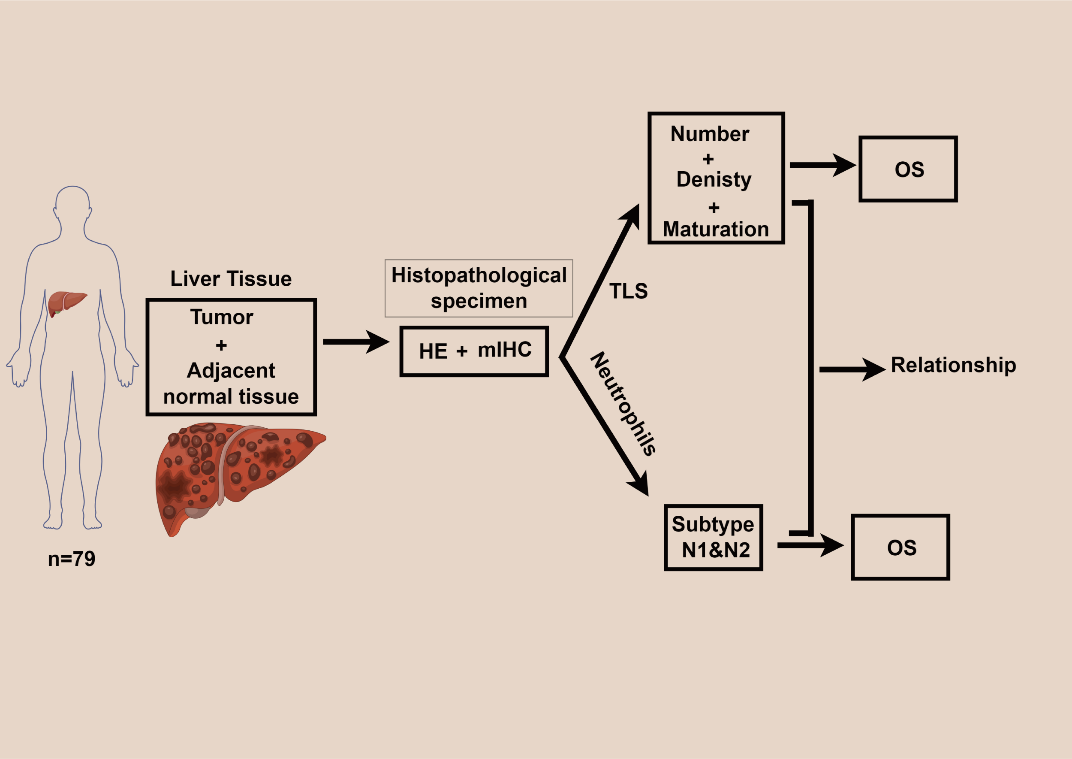


**Fig. S2:**


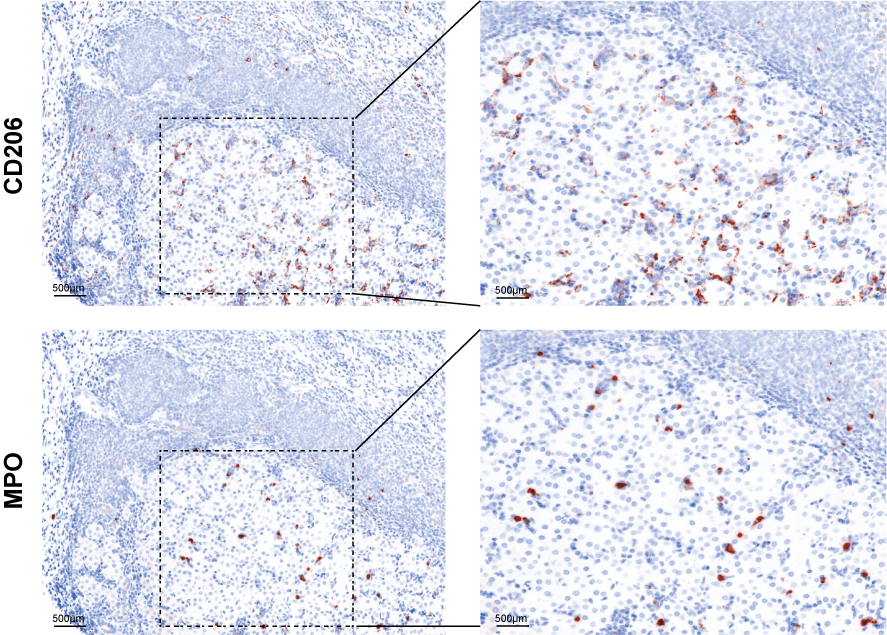


**Fig. S3:**


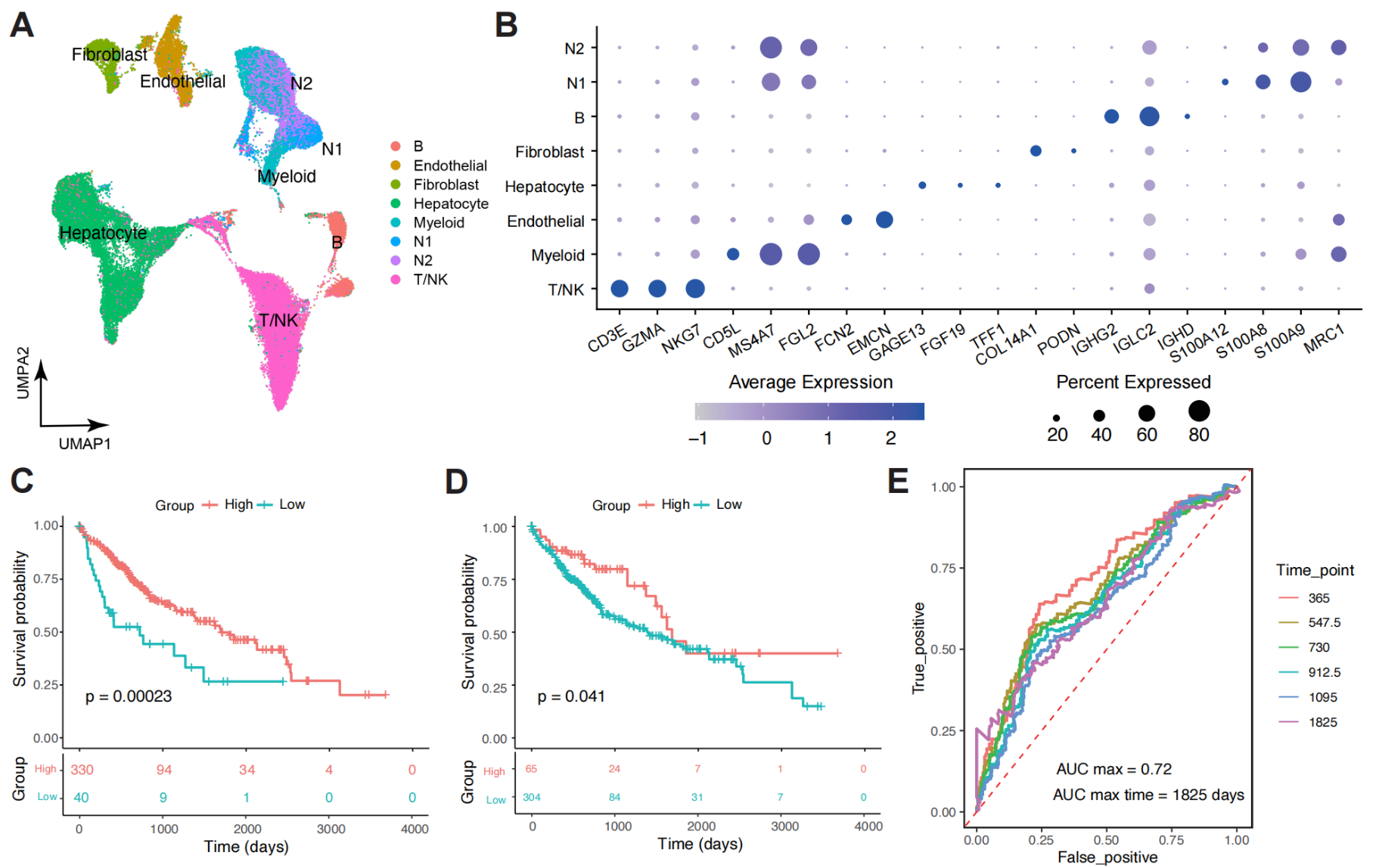


**Fig. S4:**


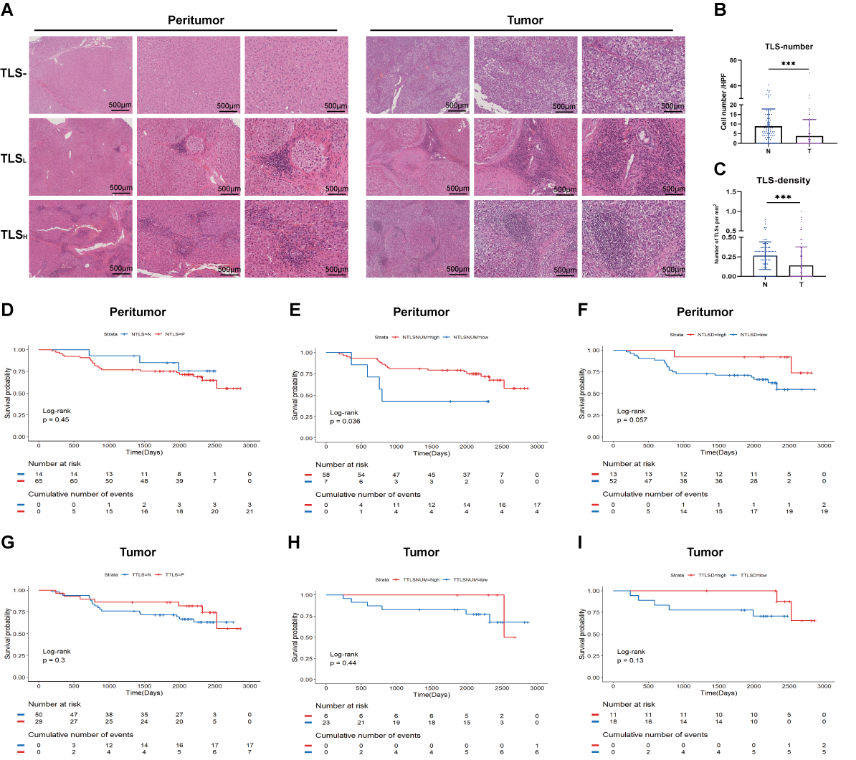


**Fig. S5:**


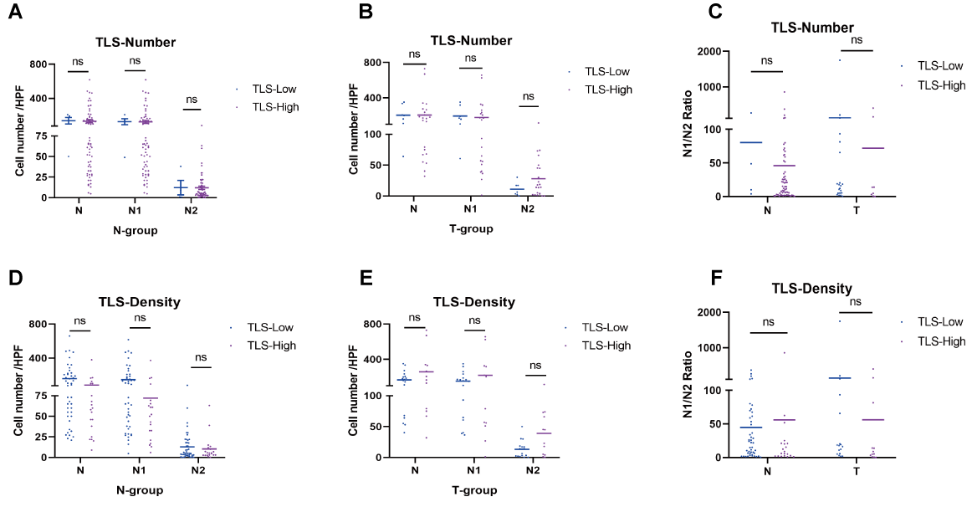


**Fig. S6:**


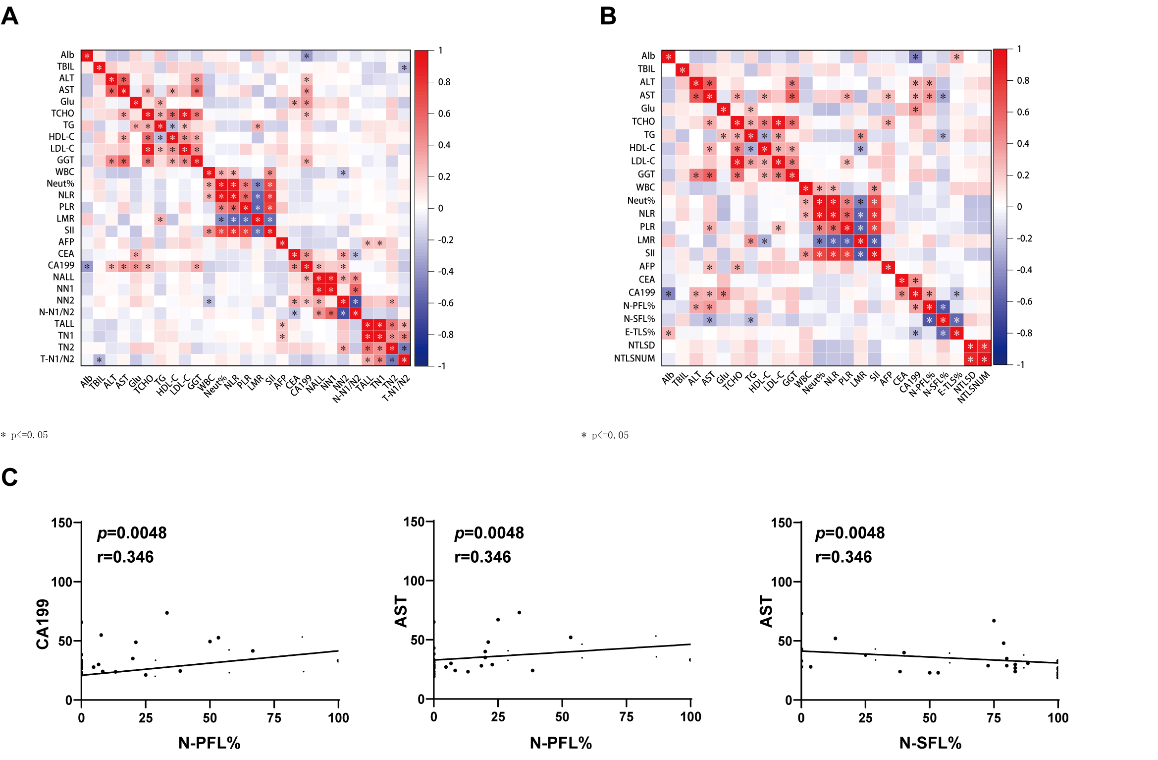


**Table S1:**

**The patient's baseline characteristics and peripheral blood indexes were expressed as medians, and interquartile ranges (IQRs) for continuous variables.**

| **Clinicopathological features** | **Values** |
| --- | --- |
| **Clinical data** | **n** |
| Female/male | 8/71 |
| Age (years): <60/≥60 | 40/39 |
| Smoking history: No/yes | 47/32 |
| **Etiology of liver disease** |  |
| HBV/HCV/alcohol/others | 68/3/3/5 |
| **Tumor** | **n** |
| Tumor size (CM): <4 / ≥ | 38/41 |
| Multiple tumors: Single/multiple | 71/8 |
| Liver capsule invasion: No/yes | 65/14 |
| Vascular invasion: No/yes | 53/26 |
| Differentiation: I+II/III+IV | 56/23 |
| TNM stage: I/II+III | 48/31 |
| **Hematological examination** | **Median（IQR）** |
| WBC (10^9/L) | 5.05 (4.13-6.15) |
| Neut percent (%) | 59.10 (54.15-65.75) |
| ALB (%) | 41.50 (39.85-44.50) |
| TBIL (μmol/L) | 15.10 (11.25-19.30) |
| ALT (U/L) | 27.00 (19.00-38.00) |
| AST (μmol/L) | 30.00 (24.00-39.50) |
| GLU (μmol/L) | 4.86 (4.43-5.39) |
| TCHO (μmol/L) | 3.98 (3.37-4.62) |
| TG (μmol/L) | 0.87 (0.69-1.21) |
| HDL-C (μmol/L) | 1.07 (0.97-1.23) |
| LDL-C (μmol/L) | 2.64 (2.19-3.24) |
| GGT (U/L) | 40.50 (27.00-75.75) |
| **Tumor marker** | **Median（IQR）** |
| CEA (ng/ml) | 2.28 (1.64-3.13) |
| CA199 (U/ml) | 16.04 (9.77-24.07) |
| AFP (ng/ml) | 40.77 (4.14-334.70) |

**Table S2:**

**The number of neutrophil infiltrations in the peritumor and tumor.**

|  |  | **Sub-group** | |
| --- | --- | --- | --- |
|  | **Cut-off** | **Low** | **High** |
| **N-group (n=79)** |  |  |  |
| total-Neu (cells/HPF) | 90.4 | n = 46 | n=33 |
| N1 (cells/HPF) | 170.0 | n = 64 | n = 15 |
| N2 (cells/HPF) | 6.2 | n = 44 | n = 35 |
| N1/N2 | 34.0 | n = 57 | n = 22 |
| **T-group (n=79)** |  |  |  |
| total-Neu (cells/HPF) | 64.0 | n = 30 | n = 49 |
| N1 (cells/HPF) | 37.0 | n = 22 | n = 57 |
| N2 (cells/HPF) | 45.4 | n = 71 | n = 8 |
| N1/N2 | 110.7 | n = 70 | n = 9 |

**Table S3:**

**Relationships between N1/N2 ratio and clinicopathological characteristics for normal and tumor respectively.** Bold = p value meets the definition of statistical significance. ^a^ Fisher’s exact test. The relationships between the tumor-associated N1/N2 ratio and clinicopathological features were examined using Pearson’s χ2 test or Fisher’s exact test.

| **Variables** | **Low N-N1/N2** | **High N-N1/N2** | **P-value** | **Low T-N1/N2** | **High T-N1/N2** | **P-value** |
| --- | --- | --- | --- | --- | --- | --- |
|  | **(n=57)** | **(n=22)** |  | **(n=70)** | **(n=9)** |  |
| Female/male | 6/51 | 2/20 | 1.000^a^ | 7/63 | 1/8 | 1.000^a^ |
| Age (years): <60/≥60 | 27/30 | 13/9 | 0.350 | 35/35 | 5/4 | 1.000^a^ |
| HBV infection: No/yes | 9/48 | 2/20 | 0.718^a^ | 8/62 | 3/6 | 0.106^a^ |
| Smoking: No/yes | 32/25 | 15/7 | 0.328 | 41/29 | 6/3 | 0.732^a^ |
| Alcohol: No/yes | 20/37 | 10/12 | 0.395 | 26/44 | 4/5 | 0.724^a^ |
| Tumor size (CM): <4/≥4 | 30/27 | 8/14 | 0.195 | 32/38 | 6/3 | 0.300^a^ |
| Multifocal: single/multiple | 52/5 | 19/3 | 0.679^a^ | 62/8 | 9/0 | 0.586^a^ |
| Liver capsule invasion: No/yes | 46/11 | 19/3 | 0.746^a^ | 57/13 | 8/1 | 1.000^a^ |
| Vascular invasion: No/yes | 37/20 | 16/6 | 0.508 | 46/24 | 7/2 | 0.710^a^ |
| Differentiation: I+II/III+IV | 39/18 | 17/5 | 0.438 | 49/21 | 7/2 | 1.000^a^ |
| TNM stage: I/II+III | 34/23 | 14/8 | 0.745 | 41/29 | 7/2 | 0.470^a^ |

**Table S4:**

**The number of TLSs’ number and density in the peritumor and tumor.**

|  |  | | **Sub-group** | |
| --- | --- | --- | --- | --- |
|  | | **Cut-off** | **Low** | **High** |
| **N-group (n=65)** | |  |  |  |
| TLS-number  (cells/HPF) | | 15.0 | n = 7 | n = 58 |
| TLS-density  (number of TLSs per mm^2^) | | 0.42 | n = 52 | n = 13 |
| **T-group (n=29)** | |  |  |  |
| TLS-number  (cells/HPF) | | 3.0 | n = 23 | n = 6 |
| TLS-density  (number of TLSs per mm^2^) | | 0.37 | n = 18 | n = 11 |

**Table S5:**

**The number of TLS% in the peritumor and tumor.**

|  |  | | **Sub-group** | |
| --- | --- | --- | --- | --- |
|  | | **Cut-off** | **Low** | **High** |
| **N-group (n=65)** | |  |  |  |
| E-TLS% (%) | | 40.0 | n = 49 | n = 16 |
| PFL-TLS% (%) | | 53.3 | n = 54 | n = 11 |
| SFL-TLS% (%) | | 22.2 | n = 23 | n = 42 |
| **T-group (n=29)** | |  |  |  |
| E-TLS% (%) | | 50.0 | n = 26 | n = 3 |
| PFL-TLS% (%) | | 40.0 | n = 25 | n = 4 |
| SFL-TLS% (%) | | 20.0 | n = 6 | n = 23 |

**Table S6:**

**Relationships between PFL% and SFL% and clinicopathological characteristics.**

| **Variables** | **Low N-PFL%** | **High N-PFL%** | **P-value** | **Low N-SFL%** | **High N-SFL%** | **P-value** |
| --- | --- | --- | --- | --- | --- | --- |
|  | **(n=54)** | **(n=11)** |  | **(n=23)** | **(n=42)** |  |
| Female/male | 7/47 | 1/10 | 1.000^a^ | 0/23 | 8/34 | **0.043^a^** |
| Age (years): <60/≥60 | 26/28 | 6/5 | 0.699 | 15/8 | 17/25 | 0.056 |
| HBV infection: No/yes | 10/44 | 0/11 | 0.190^a^ | 4/19 | 6/36 | 0.733**^a^** |
| Smoking: No/yes | 29/25 | 7/4 | 0.742^a^ | 12/11 | 24/18 | 0.700 |
| Alcohol: No/yes | 19/35 | 7/4 | 0.100^a^ | 8/15 | 18/24 | 0.525 |
| Tumor size (CM): <4/≥4 | 29/25 | 3/8 | 0.110 | 7/16 | 25/17 | **0.025** |
| Multifocal: single/multiple | 47/7 | 11/0 | 0.592^a^ | 21/2 | 37/5 | 1.000^a^ |
| Liver capsule invasion: No/yes | 45/9 | 8/3 | 0.412^a^ | 17/6 | 36/6 | 0.319^a^ |
| Vascular invasion: No/yes | 40/14 | 5/6 | 0.079^a^ | 13/10 | 32/10 | 0.100 |
| Differentiation: I+II/III+IV | 40/14 | 6/5 | 0.275^a^ | 17/6 | 29/13 | 0.680 |
| TNM stage: I/II+III | 36/18 | 5/6 | 0.304^a^ | 13/10 | 28/14 | 0.418 |

**Table S7:**

**Univariate and multivariate analysis of N1/N2 ratio and clinical prognostic parameters.**

| **Variables** | **Univariate analysis** | | **Multivariate analysis** | |
| --- | --- | --- | --- | --- |
|  | **HR (95% CI)** | **P-value** | **HR (95% CI)** | **P-value** |
| Smoking | 2.944(1.287-6.737) | **0.011** | 2.588(0.909-7.368) | 0.075 |
| Tumor size | 1.129(1.040-1.226) | **0.004** | 0.965(0.824-1.130) | 0.659 |
| Liver capsule invasion | 2.126(0.878-5.151) | 0.095 | 4.883(1.396-17.086) | **0.013** |
| Vascular invasion | 2.458(1.100-5.492) | **0.028** | 2.139(0.333-13.735) | 0.423 |
| TNM stage (I/II+III) | 2.604(1.149-5.900) | **0.022** | 1.849(0.356-9.597) | 0.464 |
| AST | 1.030(1.010-1.052) | **0.003** | 0.983(0.950-1.017) | 0.324 |
| TCHO | 1.734(1.146-2.625) | **0.009** | 1.278(0.251-6.512) | 0.767 |
| LDL-C | 1.943(1.123-3.362) | **0.018** | 1.078(0.124-9.369) | 0.946 |
| GGT | 0.007(1.005-1.010) | **0.000** | 1.008(1.003-1.013) | **0.001** |
| CA199 | 1.010(0.999-1.021) | 0.081 | 1.009(0.990-1.029) | 0.346 |
| N-N1/N2>34 | 2.303(1.013-5.236) | **0.046** | 4.484(1.550-12.966) | **0.006** |
| T-N1/N2>111 | 0.038(0.000-5.448) | 0.197 | 0(0.000-0.000) | 0.977 |

**Table S8:**

**Univariate and multivariate analysis of N-PFL%, N-SFL%, and clinical prognostic parameters.**

| **Variables** | **Univariate analysis** | | **Multivariate analysis** | |
| --- | --- | --- | --- | --- |
|  | **HR (95% CI)** | **P-value** | **HR (95% CI)** | **P-value** |
| Tumor size | 1.172 (1.069-1.285) | **0.001** | 1.093(0.937-1.275) | 0.258 |
| Liver capsule invasion | 2.643(1.060-6.588) | **0.037** | 3.737(1.225-11.400) | **0.021** |
| Vascular invasion | 4.212(1.762-10.071) | **0.001** | 1.411(0.121-16.490) | 0.784 |
| TNM stage (I/II+III) | 3.715(1.529-9.041) | **0.004** | 2.386(0.242-23.524) | 0.456 |
| AST | 1.025(1.002-1.048) | **0.030** | 0.973(0.934-1.015) | 0.202 |
| TCHO | 1.556(0.978-2.477) | 0.062 | 1.318(0.314-5.525) | 0.706 |
| LDL-C | 1.855(1.008-3.412) | **0.018** | 0.861(0.140-5.311) | 0.872 |
| GGT | 1.008(1.004-1.012) | **0.000** | 1.011(1.006-1.016) | **0.000** |
| N-PFL%>54 | 2.687(1.003-7.194) | **0.049** | 1.071(0.283-4.053) | 0.920 |
| N-SFL%>23 | 0.376(0.157-0.898) | **0.028** | 0.374(0.113-1.240) | 0.108 |

**Supplementary figure legends:**

**Fig. S1:**

**The experiment workflow for the whole study.**

The tumors and adjacent normal tissues (peritumoral tissue) obtained from 79 patients with hepatocellular carcinoma (HCC) were obtained intraoperatively. Hematoxylin and eosin (HE) staining was used to analyze the tumor lymphocytic structures (TLS), and multiplex immunohistochemistry (mIHC) staining was further used to analyze their quantity, density, and maturity, as well as N1/N2 neutrophils, and their relationship with prognosis. Additionally, the relationship between these factors and prognosis was examined, and a correlation analysis was conducted to explore the relationship between TLS and neutrophils. OS: overall survival.

**Fig. S2:**

**IHC assay to evaluate aggregates of the neutrophil**.

The specific expression of CD206 and MPO antibodies has been accurately confirmed through immunohistochemical methods. The brown portions showed positive expression of these antibodies in tissues.

**Fig. S3：**

**The biological characteristics of cells of HCC samples by single cell data analysis.**

(A) Single-cell data from GSE149614. (B) Visualization of marker gene expression. (C) Prognostic survival analysis of N1/N2 ratio in HCC tumor. (D) Relationship between TLS score and survival in HCC tumor. (E) Univariate Cox regression model using N1/N2 ratio for prognosis prediction in HCC tumor.

**Fig. S4:**

**Histological verification and prognostic value of tertiary lymphoid structures in peritumor and tumor.**

1. TLS was recognized by H&E (×4, ×10, ×20). TLS−, No aggregate in the whole slide; the best cut-off values of the total area of immune aggregation distinguish TLSL and TLSH. Scale bar=50μm/100μm/500μm
2. A comparison of the numbers of total TLSs in HCC peritumor and tumor by unpaired analysis.
3. A comparison of the density of TLSs in HCC peritumor and tumor by unpaired analysis.

(D-F) Kaplan-Meier plots for OS according to TLS in peritumor.

(G-I) Kaplan-Meier plots for OS according to TLS in the tumor.

TLS, tertiary lymphoid structure; TLSH, TLS high; TLSL, TLS low. TLS=P, TLS positive; TLS=N, TLS negative; TLS=high, TLS high; TLS=low, TLS low. OS: overall survival. ****P* < 0.001.

**Fig. S5:**

**Correlation analysis of TLS and neutrophils in peritumor and tumor.**

1. C) Comparison of different total neutrophils, N1, and N2 in peritumor and/or tumor within TLS number high and low groups.
2. F) Comparison of different total neutrophils, N1, and N2 in peritumor and/or tumor within TLS density high and low groups.
3. TLS, early TLS; PFL-TLS, primary follicle-like TLS; SFL-TLS, secondary follicle-like TLS; CD, cluster of differentiation. N, total neutrophils; N-group, peritumoral group; T-group, tumoral group; ns, no significance.

**Fig. S6:**

**Correlation analysis of the N1 and N2 neutrophils, PFL/SFL-TLS%, and clinical features.**

1. The correlation between the N1/N2 ratio and serum indexes by Spearman analysis.
2. The correlation between TLS and serum indexes by Spearman analysis.
3. Linear regression analyses between N-PFL% and CA199 level.
4. Linear regression analyses between N-PFL% and AST level.
5. Linear regression analyses between S-PFL% and AST level.

ALB, albumin; TBIL, Total Bilirubin; ALT, alanine transaminase; AST, aspartate transaminase; GLU, glucose; TCHO, total cholesterol; TG, triglyceride; HDL-C, high-density lipoprotein cholesterol; LDL-C, ow-density lipoprotein cholesterol; GGT, glutamyl transpeptidase; Neut%, the proportion of neutrophils; NLR, neutrophil-to-lymphocyte ratio; PLR, Platelet to lymphocyte ratio; LMR, Lymphocyte to monocyte ratio; SII, Systemic immune inflammation index; AFP, alpha‐fetoprotein; CEA, carcinoembryonic antigen; CA19-9, cancer antigen 19-9; NALL, total neutrophils in peritumor; NN1, tumor-associated N1 neutrophils in peritumor; NN2, tumor-associated N2 neutrophils in peritumor; N-N1/N2, N1/N2 ratio in peritumor; TALL, total neutrophils in tumor; TN1, tumor-associated N1 neutrophils in tumor; TN2, tumor-associated N2 neutrophils in tumor; T-N1/N2, N1/N2 ratio in tumor; N-PFL%, the proportion of PFL-TLS in peritumor; N-SFL, the proportion of SFL-TLS in peritumor; N-TLSD, the density of TLS in peritumor; NTLSNUM, the number of TLS in peritumor.

**Supplementary table legends:**

**Table S1:**

**The patient's baseline characteristics and peripheral blood indexes were expressed as medians, and interquartile ranges (IQRs) for continuous variables.** Abbreviations: HBV, hepatitis B virus; HCV, hepatitis C virus; TNM, tumor‐node‐metastasis; ALB, albumin; TBIL, Total Bilirubin; ALT, alanine transaminase; AST, aspartate transaminase; GLU, glucose; TCHO, total cholesterol; TG, triglyceride; HDL-C, high-density lipoprotein cholesterol; LDL-C, ow-density lipoprotein cholesterol; GGT, glutamyl transpeptidase; CEA, carcinoembryonic antigen; CA19-9, cancer antigen 19-9; AFP, alpha‐fetoprotein.

**Table S2:**

**The number of neutrophil infiltrations in the peritumor and tumor.** The median number of N1 and N2 neutrophils in TME were 72 (IQR 36.1–191.8) and 5.6 (IQR 1.6–18.3), respectively. N-group, peritumoral group; T-group, tumoral group; Neu, neutrophils; N1: N1 neutrophil; N2: N2 neutrophil; N1: N1/N2: N1/N2 ratio.

**Table S3:**

**Relationships between N1/N2 ratio and clinicopathological characteristics for normal and tumor respectively.** Bold = p value meets the definition of statistical significance. ^a^ Fisher’s exact test. The relationships between the tumor-associated N1/N2 ratio and clinicopathological features were examined using Pearson’s χ2 test or Fisher’s exact test.

**Table S4:**

**The number of TLSs’ number and density in the peritumor and tumor.** N-group, peritumoral group; T-group, tumoral group; TLS, tertiary lymphoid structures.

**Table S5:**

**The number of TLS% in the peritumor and tumor.** N-group, peritumoral group; T-group, tumoral group; E-TLS%, the proportion of early TLS; PFL-TLS%, the proportion of primary follicle-like TLS; SFL-TLS%, the proportion of secondary follicle-like TLS; TLS, tertiary lymphoid structures.

**Supplementary Table S6:**

**Relationships between PFL% and SFL% and clinicopathological characteristics.** Bold = p value meets the definition of statistical significance. ^a^ Fisher’s exact test.

The relationships between the PFL/SFL-TLS% and clinicopathological features were examined using Pearson’s χ2 test or Fisher’s exact test.

**Table S7:**

**Univariate and multivariate analysis of N1/N2 ratio and clinical prognostic parameters.** Abbreviations: AST, aspartate transaminase; TCHO, total cholesterol; LDL-C, low-density lipoprotein cholesterol; GGT, glutamyl transpeptidase; CA19-9, cancer antigen 19-9; N: peritumor; T: tumor.

**Table S8:**

**Univariate and multivariate analysis of N-PFL%, N-SFL%, and clinical prognostic parameters.** Abbreviations: AST, aspartate transaminase; TCHO, total cholesterol; LDL-C, low-density lipoprotein cholesterol; GGT, glutamyl transpeptidase; N-PFL%: the PFL-TLS% in the peritumor group; N-SFL%: the SFL-TLS% in the peritumor group.
